# Glutathione Transferase P1 Polymorphism Might Be a Risk Determinant in Heart Failure

**DOI:** 10.1155/2019/6984845

**Published:** 2019-06-02

**Authors:** Dejan Simeunovic, Natalija Odanovic, Marija Pljesa-Ercegovac, Tanja Radic, Slavica Radovanovic, Vesna Coric, Ivan Milinkovic, Marija Matic, Tatjana Djukic, Arsen Ristic, Dijana Risimic, Petar Seferovic, Tatjana Simic, Dragan Simic, Ana Savic-Radojevic

**Affiliations:** ^1^Clinic of Cardiology, Clinical Center of Serbia, 8 Koste Todorovića, 11000 Belgrade, Serbia; ^2^Faculty of Medicine, University of Belgrade, 8 Doktora Subotica, 11000 Belgrade, Serbia; ^3^Section of Cardiovascular Medicine, Department of Internal Medicine, Yale School of Medicine, New Haven, Connecticut, USA; ^4^Institute of Medical and Clinical Biochemistry, 2 Pasterova, 11000 Belgrade, Serbia; ^5^University Clinical Hospital Center “Dr. Dragisa Misovic-Dedinje”, Heroja Milana Tepica 1, 11000 Belgrade, Serbia; ^6^Clinic of Ophthalmology, Clinical Center of Serbia, 2 Pasterova, 11000 Belgrade, Serbia

## Abstract

Disturbed redox balance in heart failure (HF) might contribute to impairment of cardiac function, by oxidative damage, or by regulation of cell signaling. The role of polymorphism in glutathione transferases (*GSTs*), involved both in antioxidant defense and in regulation of apoptotic signaling pathways in HF, has been proposed. We aimed to determine whether GST genotypes exhibit differential risk effects between coronary artery disease (CAD) and idiopathic dilated cardiomyopathy (IDC) in HF patients. *GSTA1*, *GSTM1*, *GSTP1*, and *GSTT1* genotypes were determined in 194 HF patients (109 CAD, 85 IDC) and 274 age- and gender-matched controls. No significant association was found for *GSTA1*, *GSTM1*, and *GSTT1* genotypes with HF occurrence due to either CAD or IDC. However, carriers of at least one variant *GSTP1*∗Val (rs1695) allele were at 1.7-fold increased HF risk than *GSTP1*∗Ile/Ile carriers (*p* = 0.031), which was higher when combined with the variant *GSTA1*∗B allele (OR = 2.2, *p* = 0.034). In HF patients stratified based on the underlying cause of disease, an even stronger association was observed in HF patients due to CAD, who were carriers of a combined *GSTP1*(rs1695)/*GSTA1* “risk-associated” genotype (OR = 2.8, *p* = 0.033) or a combined *GSTP1*∗Ile/Val+Val/Val (rs1695)/*GSTP1*∗AlaVal+∗ValVal (rs1138272) genotype (OR = 2.1, *p* = 0.056). Moreover, these patients exhibited significantly decreased left ventricular end-systolic diameter compared to *GSTA1*∗AA/*GSTP1*∗IleIle carriers (*p* = 0.021). Higher values of ICAM-1 were found in carriers of the *GSTP1*∗IleVal+∗ValVal (rs1695) (*p* = 0.041) genotype, whereas higher TNF*α* was determined in carriers of the *GSTP1*∗AlaVal+∗ValVal genotype (rs1138272) (*p* = 0.041). In conclusion, *GSTP1* polymorphic variants may determine individual susceptibility to oxidative stress, inflammation, and endothelial dysfunction in HF.

## 1. Introduction

For more than a decade, it has been suggested that a complex interplay between oxidative stress and chronic inflammation represents one of the underlying mechanisms of gradual cardiac depression in heart failure (HF) [1–3]. Oxidative stress in HF is believed to be a consequence of increased circulating neurohormones and hemodynamic disorder, as well as inflammation and decreased oxygen delivery. On the other hand, disturbed redox balance in patients with HF might contribute to further impairment of cardiac function, either by oxidative damage to vital cellular molecules or by affecting cell signaling involved in cell survival and death [4]. There is overwhelming evidence for the presence of oxidative stress in all phases of HF in animal models and humans [5, 6]. Regarding the mechanisms of oxidative stress in HF, both enhanced free radical production and diminished antioxidative defense are involved in the occurrence and progression of HF [5]. It is important to note that increased free radical production and inflammation are involved in cardiomyocyte apoptosis and progression of HF. Continuous release of free radicals in response to angiotensin II and catecholamines has also been found to take part in cardiac hypertrophy. Additionally, structural changes and activation of metalloproteinases are also dependent on free radicals produced in the course of fibroblast to myofibroblast transformation. Taken together, all these free radical-dependent processes contribute to the occurrence of end-stage HF [5]. Several biomarkers of oxidative distress, such as isoprostanes, malondialdehyde, uric acid, and protein carbonyl groups, have been shown to be elevated in different stages of HF [7, 8].

In addition to this well-established link, recent findings on the adverse effect of chemical and pollutant exposure to heart disease [9, 10] put special emphasis on the role of genetic polymorphisms of enzymes involved in detoxification of xenobiotics and antioxidant defense in the HF syndrome [11]. Members of the glutathione transferase (GST) enzyme superfamily belong to phase II detoxification enzymes but are also involved in regulation of the cellular redox state through different antioxidant catalytic and noncatalytic roles [12]. Moreover, almost all members of the GST family exhibit genetic polymorphisms, which can result in a complete lack or lowering of enzyme activity [13].

Considering the fact that HF represents a multifactorial, polygenic syndrome, the role of oxidative stress and consequently polymorphic expression of GSTs may have a different impact, especially regarding the specific cause of heart failure. In coronary artery disease (CAD) as the most common etiology of heart failure in industrialized countries, genetic epidemiologic studies mostly investigated the association of common *GSTA1*, *GSTM1*, *GSTT1*, and *GSTP1* polymorphisms with disease risk [14–16]. Among them, the most attention was focused on the investigation of *GSTM1* and *GSTT1* deletion polymorphisms [17], considering the fact that the homozygous deletions of these genes result in a complete lack of enzymatic activity and thus diminish detoxification capacity [18]. Based on the important role of the GSTM1 enzyme in detoxifying benzodiolepoxide, present in tobacco smoke and environmental pollution, it could be speculated that carriers of the *GSTM1*-null genotype could have increased risk of CAD, particularly in smokers. Until now, the results on the independent effect of the *GSTM1*-null genotype on increased susceptibility to CAD are still being debated [14, 17]. On the other hand, the recent meta-analysis involving 47596 subjects showed that the *GSTM1-null* genotype in association with smoking increases the risk for CAD [19]. Moreover, correlation between the *GSTM1-null* genotype and indices of inflammation and oxidative stress has been demonstrated in CAD. Thus, higher CRP and lower total antioxidant capacity have been observed in CAD patients lacking *GSTM1* than those with an active GSTM1 enzyme [20]. With regard to the *GSTT1-null* genotype, only few studies revealed that the *GSTT1-null* genotype carries higher risk for HF development [14, 17]. Two genetic variants in the *GSTP1* gene, the *GSTP1*∗G allele (rs1695) coding for protein in which amino acid isoleucine (Ile) is substituted with valine (Val) at position 105 and the *GSTP1*∗ allele (rs1138272) in which alanine (Ala) is substituted with (Val) at position 114, have been shown to confer altered catalytic and noncatalytic activity, whereas the *GSTA1*∗B allele (rs3957356) is associated with the lower expression of *GSTA1* than that of the common *GSTA1*∗A allele. It seems reasonable to assume that *GSTA1*- or *GSTP1-variant* genotypes also might contribute to the endogenous predisposition to oxidative damage in the setting of disrupted redox balance in HF patients due to CAD. However, the results of association of *GSTP1* and *GSTA1* polymorphisms with risk for CAD are still inconsistent [14, 21]. Interestingly, in idiopathic dilated cardiomyopathy (IDC), as a rare entity of HF syndrome, the effect of genetic polymorphisms of these enzymes has still not been investigated.

Having all that in mind, we conducted a pilot case-control study consisting of patients with HF due to coronary artery disease (CAD) or idiopathic dilated cardiomyopathy (IDC) in order to compare the distribution of common GST genotypes and the differential risk effect between these two entities.

## 2. Materials and Methods

### 2.1. Subjects

A total of 194 patients (51 women and 143 men, all Caucasian) with HF were enrolled in the study. We included two kinds of patients in the study: 109 of those with HF due to coronary artery disease (CAD) and 85 of those with heart failure due to idiopathic dilated cardiomyopathy (IDC). All patients were recruited between 2008 and 2012 from the Medical Center “Bezanijska Kosa” and from the Clinical Center of Serbia, during the dispensary checkups. Diagnosis of HF was based on the patient's history, physical examination, electrocardiography, chest X-ray, echocardiography, and coronary angiography. Distribution of the New York Heart Association (NYHA) stage was indicated for HF patients (Table 1). Major inclusion criteria were the left ventricular ejection fraction < 45% and stable HF over the two weeks prior to enrollment. For the CAD subpopulation, the inclusion criterion was evidence of CAD on angiography. For the IDC subpopulation, major inclusion criteria were the absence of CAD on coronary angiography and the evidence of chamber dilation. Patients with congenital, acquired valvular, or pericardial abnormalities were excluded from the study. Our case-control study also included a total of 274 individuals in the control group. The study was approved by the ethics committee of the Clinical Center of Serbia (470/XII-9 from 29/12/2008), and all study participants signed an informed consent.

### 2.2. *GST* Genotyping

Genomic DNA was isolated from whole blood using the QIAGEN QIAamp kit (QIAGEN Inc., Chatsworth, CA). *GSTA1* (-69C>T) and *GSTP1* (Ile105Val, rs1695) were examined by the polymerase chain reaction-restriction fragment length polymorphism (PCR-RFLP) method [22, 23], whereas the *GSTM1/GSTT1* were determined by the PCR method [24]. The *GSTP1* (Ala114Val, rs1138272) polymorphism was determined by qPCR (Applied Biosystems) only in CAD patients using an Applied Biosystems TaqMan Drug Metabolism Genotyping Assay (ID C_1049615_20). The primer sequences, PCR conditions, restriction enzymes used, and respective restriction conditions, as well as fragment lengths after electrophoresis on 2% agarose gel, can be found in Table 2.

### 2.3. Determination of Parameters of Inflammation, Oxidative Stress, and Endothelial Dysfunction in Plasma/Serum

Malondialdehyde (MDA) was determined spectrophotometrically (BIOXYTECH LPO-586 kit; OxIS Research, Portland, OR, USA). The results were expressed in *μ*mol/L. Serum levels of hs-CRP were determined using a commercially available kit. Commercially available ELISA kits for TNF*α*, ICAM-1, VCAM-1 (Bender MedSystems, GmbH, Austria) were used for the measurement of those inflammatory markers in plasma/serum samples collected from each patient.

### 2.4. Noninvasive Assessment of Endothelium-Dependent and Endothelium-Independent Flow-Mediated Dilation (FMD) of the Brachial Artery

Endothelium-dependent and endothelium-independent FMD of the brachial artery was assessed by a 13.0 MHz linear array transducer (Vivid 7, GE Medical Systems, Little Chalfont, England, UK) as previously published.

### 2.5. Statistical Analysis

In descriptive statistics, we summarized all continuous variables by means ± standard deviations (SD). Differences in investigated parameters were assessed by using analysis of variance (ANOVA) and Student's t-test for continuous variables and *χ*^2^ for categorical variables. The associations between the genotypes and HF risk were calculated by using logistic regression to compute odds ratios (ORs) and corresponding 95% confidence intervals (CIs), adjusted according to age, gender, smoking, hypertension, and diabetes as potential confounding factors. Haplotype analysis of *GSTP1* SNPs was examined using the SNPStats. In order to demonstrate the validity of our data, a positive control was introduced by assessing the well-established association between *GSTM1* deletion polymorphism in smokers and the risk of bladder cancer [25, 26]. For this purpose, an adjusted OR to age, gender, and BMI was calculated. For the data with a nonnormal distribution, we used the Mann-Whitney rank-sum test for between-two-group comparisons. Two-tailed *p* values of <0.05 were considered significant. Data were analyzed using the Statistical Package for the Social Sciences (SPSS) (version 17.0, Chicago, IL).

## 3. Results

### 3.1. General Characteristics of Patients

The characteristics of the whole group of HF patients, as well as patients stratified to underlying disease due to IDC and CAD, along with the characteristics of control participants are shown in Table 1. While the average age of both the control group and the HF patients is around 55 years, patients with IDC appear to be 6 years younger on average (mean age = 49.0) and thus statistically differ from the remaining two groups, which is consistent with findings in the literature [27, 28]. The occurrence of diabetes and hypertension was significantly higher in patients and patient subpopulations compared to controls. Gender and smoking status did not differ significantly between HF patients and controls.

### 3.2. Distribution of GST Genotypes

The distribution of GST genotypes in all HF patients and controls is shown in Table 3. The frequencies of the GSTA1, GSTP1, GSTM1, and GSTT1 genotypes are in accordance with the reported values in the literature. In order to fully estimate the role of GST genotypes in HF development, respected ORs were adjusted to factors regarded as confounding variables: age, gender, smoking, hypertension, and diabetes. Significant association between the GST genotype and the risk of HF development was found only for the GSTP1 genotype (rs1695) (Table 3). Namely, the risk of HF was significantly higher in individuals with at least one variant, ∗Val allele, i.e., GSTP1∗Ile/Val or GSTP1∗Val/Val genotypes (OR = 1.7; 95%CI = 1.0 − 2.9; p = 0.031). The observed association to HF risk was even more potentiated when the risk-associated GSTP1 genotype was combined with at least one variant GSTA1 allele (OR = 2.2; 95%CI = 1.1 − 4.4; p = 0.034). On the other hand, data validity assessment demonstrated a 4.4-fold elevated risk of bladder cancer in smokers with the GSTM1-null genotype as opposed to GSTM1-active nonsmokers.

Patients with HF due to CAD and IDC demonstrated similar GST genotype distribution. No significant association was observed for GSTA1, GSTM1, and GSTT1 genotypes with the occurrence of HF due to either CAD or IDC. When we analyzed the effect of GSTP1 genotypes (rs1695 and rs1138272) according to the specific cause of HF, a 1.9-fold increased risk was observed for carriers of at least one GSTP1∗Val (rs1695) allele in the CAD group (95%CI = 1.0 − 3.6; p = 0.056) (Table 4(a)). Patients from the IDC group with the same genotype were at 1.4-fold risk of HF (95%CI = 0.7 − 2.7; p = 0.284) (Table 4(b)). Namely, although these results did not reach the level of statistical significance, the evident risk-associated trend was observed. However, combined GSTA1∗AB+BB/GSTP1∗IleVal+∗ValVal (rs1695) and GSTA1∗AB+BB/GSTP1∗AlaVal+∗ValVal (rs1138272) genotypes potentiated the risk in HF patients due to CAD (OR = 2.8, 95%CI = 1.1 − 7.3, p = 0.033, and OR = 2.1, 95%CI = 1.0 − 4.5, p = 0.056, respectively). Moreover, we performed haplotype analysis generating four GSTP1 haplotypes: wild-type GSTP1∗A (Ile105/Ala114), GSTP1∗B (Val105/Ala114), GSTP1∗C (Val105/Val114), and GSTP1∗D (Ile105/Val114). Haplotype analysis conferred small yet nonsignificant risk for CAD-related HF development in the case of the GSTP1∗B haplotype (OR = 1.4; 95%CI = 0.9 − 2.3; p = 0.170; Table 5). On the other hand, GSTP1∗C and GSTP1∗D haplotypes exhibited lower risk towards CAD-related HF development (OR = 0.5, 95%CI = 0.1 − 3.9, p = 0.490, and OR = 0.6, 95%CI = 0.1 − 3.1, p = 0.560, respectively; Table 5).

### 3.3. Risk-Associated *GST* Genotypes in relation to the Parameters of Oxidative Stress, Inflammation, and Endothelial Dysfunction in the CAD-Related HF Subgroup

Plasma levels of MDA, end product of lipid peroxidation, and established marker of lipid oxidative damage were analyzed in CAD-related HF patients stratified according to polymorphism in GSTA1 and GSTP1 antioxidant enzymes (Table 6). No significant difference was observed in MDA levels between carriers of either GSTA1 or GSTP1 (rs1695 and rs1138272) genotypes in the CAD subgroup. Moreover, inflammatory markers TNF*α* and hs-CRP together with biochemical markers of endothelial dysfunction, ICAM-1 and VCAM-1, were also stratified according to GST risk genotypes. The results have shown that higher values of ICAM-1 were found in carriers of GSTP1∗IleVal+∗ValVal (rs1695) (*p* = 0.041), whereas higher TNF*α* was present in carriers of GSTP1∗AlaVal+∗ValVal (rs1138272) (*p* = 0.041) (Table 6).

### 3.4. The Association of *GSTP1* Genotype with the Indices of HF Severity

The role of *GSTP1* polymorphism was further analyzed regarding parameters related to the severity of HF. The dimensions of the left ventricle after systole and diastole (LVESD and LVEDD), along with NO-dependent and NO-independent vasodilation of the brachial artery in CAD patients with different *GSTP1* genotypes, are shown in Table 7. The end-systolic (LVESD) and end-diastolic (LVEDD) diameters of the left ventricle did not differ significantly between patients with different *GSTP1* genotypes (rs1695 and rs1138272). Likewise, the degree of endothelium-dependent NO-mediated vasodilation and endothelium-independent nitroglycerin- (NTG-) mediated vasodilation of the brachial artery was similar between CAD-related HF patients with either the *GSTP1* wild-type genotype and carriers of at least one variant *GSTP1* allele. When we analyzed these parameters in CAD-related HF patients stratified according to combined *GSTA1/GSTP1* (rs1695) genotypes (Table 7), only carriers of variant *GSTA1*∗B/*GSTP1*∗Val (rs1695) alleles had significantly decreased LVESD compared to individuals with *GSTA1*∗AA/*GSTP1*∗IleIle (*p* = 0.021).

LVEDD: left ventricular end-diastolic diameter; LVESD: left ventricular end-systolic diameter; FMD-NO: endothelium-dependent NO-mediated vasodilation; FMD-NTG: endothelium-independent NTG-mediated vasodilation. ^a^Student *t*-test was used for testing differences in LVEDD and LVESD, for each group compared to the reference group (*GSTP1*∗Ile/Ile or combined *GSTA1/GSTP1*∗AA/∗IleIle , *GSTA1/GSTP1*∗AA/∗AlaAla). ^b^Mann-Whitney test was used for testing differences in FMD-NO and FMD-NTG, for each group compared to the reference group (*GSTP1*∗Ile/Ile or combined *GSTA1/GSTP1*∗AA/∗IleIle, *GSTA1/GSTP1*∗AA/∗AlaAla). ^∗^Statistically significant at *p* < 0.05.

## 4. Discussion

Based on different roles of GSTs and considering the fact that in the setting of heart failure the disturbances of redox regulation can contribute to disease progression, in this study, we investigated the effect of common *GST* polymorphisms regarding specific HF entities. Among tested *GST* polymorphisms, only the variant *GSTP1*∗Val allele has shown a significant association with HF, regardless of the specific cause. This HF risk conferred by *GSTP1* polymorphism was even higher when combined with the variant *GSTA1*∗B allele. In HF patients stratified according to the underlying cause of the disease, even more potentiated association was observed in HF patients due to CAD, while in those due to idiopathic cardiomyopathy, despite the evident trend, this association was not confirmed.

In our study, we found no significant association for individual GSTM1 and GSTT1 polymorphisms with the occurrence of HF due to either CAD or IDC. Our results are in concordance with the study of Norskov et al., who conducted a comprehensive analysis of the copy number variation for GSTM1 and GSTT1 in patients with ischemic heart disease and ischemic cerebrovascular disease, showing no significant association with disease risk, even among smokers.

It has been well established that GSTP1 exhibits both antioxidant and glutathionylation activity, having important role in the maintenance of the cellular redox state [29]. Namely, GSTP1 is necessary for the activation of peroxiredoxin VI (Prdx6), a member of the family of antioxidant enzymes, which catalyzes detoxification of lipid peroxides, particularly in biological membranes [30]. After the exposure of endothelial cells to laminar shear stress, as a result of increase in free radical production, the upregulation of these antioxidant enzymes has been observed, probably as adaptive phenomenon [31]. Even more, their important role in regulating endothelial cell activation during atherosclerosis has been proposed. The most recent data on MCF-7 cells showed that the polymorphic expression of GSTP1 differentially interposes the Prdx6 activity, implying that depending upon their *GSTP1* genotype, individuals will have significant differences in mounting an antioxidant response [30]. In carriers of the *GSTP1* variant genotype, changed GSTP1 catalytic activity could deepen the progression of the disease, which consequently results in multiple cellular responses, such as DNA synthesis, transcription factor activation, and alteration of protein expression. If these results are translated to the HF setting, it may be speculated that *GSTP1*∗Ile/Ile carriers might possibly have a higher antioxidant potential providing the favorable environment for better prognosis. Moreover, it is important to note that the highest HF risk was found for carriers of combined *GSTA1*∗B*/GSTP1*∗Val variant alleles. Namely, GSTA1-1 is one of the most promiscuous GST enzymes with wide substrate specificity, including powerful antioxidant activity [32]. Thus, the presence of the *GSTA1*∗B gene variant, which results in lower expression of the enzyme, in combination with the *GSTP1*∗Val allele, might significantly contribute to decreased antioxidant capacity of HF patients, carriers of the combined *GSTA1*∗B*/GSTP1*∗Val genotype. Regarding our previous data on the prognostic significance of oxidative stress and inflammatory parameters in HF [3, 7], we investigated whether *GSTA1* and *GSTP1* polymorphic variants could affect the plasma concentration of MDA, TNF*α*, and hs-CRP in our cohort of CAD-related HF patients. Indeed, we showed that carriers of the variant *GSTP1*∗Val (rs1138272) genotype demonstrated higher TNF*α* levels, revealing new functional relevance of this *GSTP1* polymorphism.

Aside from generation of reactive oxygen species (ROS) in the failing myocardium, endothelial activation also significantly contributes to myocyte apoptosis, necrosis, and remodeling of the extracellular matrix in the heart [33]. GSTP1 participates in regulation of stress signaling and apoptosis via its noncatalytic activity. Specifically, through protein:protein interaction, GSTP1 acts as an endogenous inhibitor of several signaling molecules, including c-Jun N-terminal kinase (JNK) [34] and TNF*α* receptor-associated factor 2 (TRAF2) [35]. This interaction of GSTP1 with the MAPK and NF-*κ*B axes of regulation is also responsible for its suggested anti-inflammatory role [36]. What is more, the degree of interaction between GSTP1 and JNK, a member of the mitogen-activated protein kinase (MAPK) signaling pathway, depends on the redox status of the cell. In that way, GSTP1 provides an important link between cellular redox potential and the regulation of kinase pathways involved in apoptosis and inflammation. The results of Andrukhova et al., showing elevated GSTP1 expression in the failing myocardium, were associated with reduced GSTP1:JNK interaction and consequent activation of the JNK-MAPK signaling cascade, essential for cardiomyocyte apoptosis [37], representing further confirmation of the contributing role of oxidative stress in the HF progression. Interestingly, the same authors indicated that a single dose of recombinant GSTP1 has cardioprotective effect in rats after myocardial infarction, affecting both inflammatory and apoptotic responses [37]. The substitutions of amino acid isoleucine (Ile) with valine (Val) at position 105 and alanine (Ala) with (Val) at position 114 as a consequence of *GSTP1* polymorphisms can also affect the aforementioned interaction with JNK, causing an alteration in the GSTP1-mediated inhibitory effect of JNK activity [38]. Indeed, it has been shown that the *GSTP*∗C (Val105/Val114) haplotype is a more potent JNK inhibitor than the referent *GSTP1*∗A (Ile105/Ala114) [38]. Although we did not find significant association between different GSTP1 haplotypes and the CAD-related HF risk, the obvious trend for decreasing HF risk in carriers of the *GSTP1*∗C (Val105/Val114) haplotype might have a molecular explanation in its ability to prevent apoptosis more efficiently. A further indication of functional GST redundancy is provided by the fact that GSTA1 and GSTM1 were also capable of associating with JNK. Based on our results on increased disease risk for HF patient carriers of the combined *GSTA1/GSTP1* variant genotype, it might be speculated that the impaired GSTP1:JNK interaction in CAD [37] could be further modified in patient carriers of the combined *GSTA1*/*GSTP1* risk-associated genotype.

Finally, this seemingly “pleiotropic” modulatory role of GSTP1 was recently demonstrated for the key regulatory molecule of cell-cell communication in the heart, the signal transducer and activator of transcription 3 (STAT3) [39]. STAT3 is fundamental for physiological homeostasis and stress-induced remodeling of the heart, as reviewed in the article of Haghikia et al. [40]. Furthermore, Chen et al. demonstrated that GSTP1, due to its interaction with STAT3, can inhibit angiotensin II-induced STAT3 activation of vascular smooth muscle cells (VSMCs) *in vitro* [39], thus preventing VSMC proliferation. However, the potential effect of *GSTP1* polymorphism on this interaction still remains elusive.

Based on recent findings on increased GSTP1 catalytic activity in the metabolism of polycyclic aromatic hydrocarbons from tobacco smoke in carriers of the variant GSTP1∗Val allele, which is located in the substrate-binding site, it may be hypothesized that GSTP1 genotyping could provide additional information not exclusively regarding tobacco smoking but also to other recognized GSTP1 substrates present in air pollutants, dietary compounds, products of endogenous metabolism, and lipid peroxidation. It would be tempting to investigate whether metabolism of ubiquitous reactive aldehyde acrolein, as a typical example of such a GSTP1 substrate associated with increased cardiovascular disease risk, would be affected by GSTP1 polymorphism [41]. In conclusion, due to involvement of GSTs in detoxification of xenobiotics from tobacco smoke and also the metabolism of environmental and occupational pollutants, as well as various dietary constituents, different lifestyles might affect the role of GSTs within one population, while at the same time, the association observed in one may not be seen in other populations living in different environments.

To take further insight into molecular mechanisms and potential consequences of *GSTP1* and *GSTA1* polymorphisms with respect to CAD-related HF, we correlated the *GSTP1* and *GSTA1* variant genotypes with both the indices of heart remodeling and parameters of endothelial dysfunction. However, we found that only carriers of combined variant *GSTA1*∗B/*GSTP1*∗Val alleles had significantly decreased LVESD compared to individuals with *GSTA1*∗AA/*GSTP1*∗IleIle genotypes. Besides, the GSTP1 variant genotype was significantly associated with soluble ICAM-1 levels in these patients. As such, GSTP1 polymorphic variants may determine individual susceptibility to oxidative stress, inflammation, and endothelial dysfunction in HF, as well.

In our cohort of IDC patients, despite the evident trend, significant association of *GSTP1* polymorphism with disease risk was not confirmed. The most probable reason is the small number of patients with ICD, having in mind the findings of Inoue et al. and Cannon et al. on the presence of coronary microcirculatory dysfunction despite angiographically normal epicardial coronary arteries in these patients [42, 43]. Hence, altered coronary flow reserve (CFR), which reflects coronary microvascular function and integrity, has been reported as an independent predictor of subsequent cardiac events in patients with idiopathic LV dysfunction [44].

Certain limitations could be considered in our study. The study findings may be influenced by potential biases arising from relatively small number of participants and *GST* polymorphisms studied. The number of patients was further reduced by analyzing subgroups of HF due to CAD and IDC. Furthermore, the use of population controls may have been more appropriate. Additionally, we cannot entirely rule out the possibility that some of our results could be caused by confounding, although we adjusted all results by age, gender, smoking, hypertension, and diabetes as potential confounding factors. Moreover, despite a large number of smokers in our cohort of HF patients, the correction for continuous measure of smoking was not determined.

## 5. Conclusions

It may be concluded that the variant *GSTP1*∗Val allele is significantly associated with HF risk regardless of the specific cause. This association is even more potentiated in carriers of both *GSTP1*∗Val and *GSTA1*∗B alleles.

## Figures and Tables

**Table 1:** Selected characteristics of patients with HF and controls.

	Controls	HF	IDC	CAD
Age (years) ± SD^a^	55.8 ± 10.9	54.3 ± 10.8	49.0 ± 13.6^∗^	58.7 ± 4.3
Gender^b^				
Females (%)	90 (33)	51 (26)	15 (18)^∗^	36 (33)
Males (%)	184 (67)	143 (74)	70 (82)^∗^	73 (67)
Diabetes^b^				
Yes (*n* (%))	25 (9)	66 (35)^∗^	25 (30)^∗^	41 (38)^∗^
No (*n* (%))	249 (91)	123 (65)^∗^	57 (70)^∗^	66 (62)^∗^
Hypertension^b^				
Yes (*n* (%))	67 (26)	82 (52)^∗^	16 (19)	68 (78)^∗^
No (*n* (%))	191 (74)	77 (48)^∗^	69 (81)	19 (22)^∗^
Smoking status^b^				
Smokers (*n* (%))	138 (52)	99 (57)	42 (51)	57 (61)
Nonsmokers (*n* (%))	126 (48)	76 (43)	40 (49)	36 (39)
NYHA				
II		124 (64)	54 (64)	71 (65)
III		48 (25)	20 (24)	28 (26)
IV		18 (9)	8 (9)	10 (9)

HF: heart failure; IDC: idiopathic dilated cardiomyopathy; CAD: coronary artery disease; SD: standard deviation; NYHA: New York Heart Association functional classification of heart failure. ^a^Student's *t*-test; ^b^*χ*^2^ test; ^∗^statistically significant in comparison to controls (*p* < 0.05).

**Table 2:** PCR-RFLP: primer sequences, PCR conditions, restriction enzymes, and fragment lengths.

Polymorphism	Primer sequences	PCR protocol	Gel electrophoresis results
*GSTA1*∗C-69T	F, 5′-GCATCAGCTTGCCCTTCA -3′ R, 5′-AAACGCTGTCACCGTCCTG-3′	Denature: 94°C for 3 min Followed by 94°C for 30 s Annealing: 56°C for 30 s Extension: 72°C for 30 s #cycles: 30 Final extension: 72°C for 10 min	Eam1104I incubation at 37°C overnight *GSTA1*∗CC: 400 bp *GSTA1*∗CT: 400 bp, 308 bp, and 92 bp *GSTA1*∗TT: 308 bp and 92 bp

*GSTP1*∗Ile105Val	F, 5′-ACCCCAGGGCTCTATGGGAA-3′ R, 5′-TGAGGGCACAAGAAGCCCCT-3′	Denature: 95°C for 10 min Followed by 94°C for 30 s Annealing: 59°C for 30 s Extension: 72°C for 30 s #cycles: 29 Final extension: 72°C for 10 min	Alw26I incubation at 37°C overnight *GSTP1*∗Ile/Ile: 176 bp *GSTP1*∗Ile/Val: 176 bp, 91 bp, and 85 bp *GSTP1*∗Val/Val: 91 bp and 85 bp

*GSTM1*	F, 5′-GAACTCCCTGAAAAGCTAAAGC-3′ R, 5′-GTTGGGCTCAAATATACGGTGG-3′	Multiplex PCR: Denature: 94°C for 3 min Followed by 94°C for 30 s Annealing: 59°C for 30 s Extension: 72°C for 45 s #cycles: 30 Final extension: 72°C for 4 min	*GSTM1*∗active: 215 bp band *GSTM1*∗null: absent band
*GSTT1*	F, 5′-TTCCTTACTGGTCCTCACATCTC-3′ R, 5′-TCACGGGATCATGGCCAGCA-3′		*GSTT1*∗active: 480 bp band *GSTT1*∗null: absent band
*CYP1A1*	F, 5′-GAACTGCCACTT CAGCTGTCT-3′ R, 5′-CAGCTGCATTTG GAAGTGCTC-3′		Successful PCR reaction: 312 bp band Unsuccessful PCR reaction: absent band

**Table 3:** Distribution of *GSTA1*, *GSTP1*, *GSTM1*, and *GSTT1* genotypes in HF patients and controls.

Genotype	Controls (*n* (%))	HF patients (*n* (%))	OR (95% CI)^a^	*p*
*GSTA1*				
∗A/A	112 (41)	68 (35)	1.0^b^	
∗A/B+B/B	162 (59)	125 (65)	1.5 (0.9-2.5)	0.097
*GSTP1*∗Ile105Val (rs1695)				
∗Ile/Ile	115 (42)	68 (35)	1.0^b^	
∗Ile/Val+∗Val/Val	159 (58)	124 (65)	1.7 (1.0-2.9)	0.031^∗^
*GSTM1*				
Active	137 (50)	92 (47)	1.0^b^	
Null	137 (50)	102 (53)	1.1 (0.7-1.8)	0.671
*GSTT1*				
Active	203 (74)	146 (74)	1.0^b^	
Null	71 (26)	48 (26)	0.9 (0.5-1.7)	0.873
Combined *GSTA1/ GSTP1*(rs1695)				
∗AA/∗IleIle	54 (20)	29 (16)	1.0^b^	
∗AA/∗IleVal+∗ValVal	58 (21)	39 (20)	1.2 (0.5-2.6)	0.697
∗AB+∗BB/∗IleIle	61 (22)	39 (20)	1.0 (0.5-2.3)	0.913
∗AB+BB/∗IleVal+∗ValVal	101 (37)	84 (44)	2.2 (1.1-4.4)	0.034^∗^
GST genotype/smoking status	Controls (*n* (%))	Bladder cancerpatients (*n* (%))	OR (95% CI)^c^	*p*
*GSTM1-active/*nonsmoker	50 (46)	16 (18)	1.00^b^	
*GSTM1-null*/smoker	58 (54)	75 (82)	4.4 (2.2-8.9)	<0.001

HF: heart failure; CI: confidence interval; OR: odds ratio. ^a^Logistic regression to compute odds ratios (ORs)adjusted for gender, age, smoking, hypertension, and diabetes. ^b^Reference group. ^c^Logistic regression to compute odds ratios adjusted for gender, age, and body mass index. ^∗^Statistically significant in comparison with the reference genotype (*p* < 0.05).

**Table:** (a) Distribution of *GSTA1*, *GSTP1*, *GSTM1*, and *GSTT1* genotypes in HF patients due to CAD and controls

	Controls (*n* (%))	Patients (*n* (%))	OR (95% CI)^a^	*p*
*GSTA1*				
∗A/A	112 (41)	38 (35)	1.0^b^	
∗A/B+B/B	162 (59)	70 (65)	1.8 (0.9-3.5)	0.075
*GSTM1*				
Active	137 (50)	53 (49)	1.0^b^	
Null	137 (50)	56 (51)	1.1 (0.6-2.0)	0.749
*GSTT1*				
Active	203 (74)	82 (75)	1.0^b^	
Null	71 (26)	27 (25)	1.0 (0.5-2.0)	0.935
*GSTP1*∗Ile105Val (rs1695)				
∗Ile/Ile	115 (42)	39 (37)	1.0^b^	
∗Ile/Val+∗Val/Val	159 (58)	68 (63)	1.9 (1.0-3.6)	0.056
*GSTP1*∗Ala114Val (rs1138272)				
∗Ala/Ala	205 (85)	90 (86)	1.0^b^	
∗Ala/Val+∗Val/Val	38 (15)	15 (14)	0.5 (0.2-1.3)	0.157
Combined *GSTA1/GSTP1* (rs1695)				
∗AA/∗IleIle	54 (20)	16 (15)	1.0^b^	
∗AA/∗IleVal+∗ValVal	58 (21)	22 (21)	1.4 (0.5-4.1)	0.539
∗AB+∗BB/∗IleIle	61 (22)	23 (22)	1.4 (0.5-4.0)	0.560
∗AB+BB/∗IleVal+∗ValVal	101 (37)	45 (42)	2.8 (1.1-7.3)	0.033^∗^
Combined *GSTA1/GSTP1* (rs1138272)				
∗AA/∗AlaAla	28 (11)	9 (9)	1.0^b^	
∗AA/∗AlaVal+∗ValVal	84 (33)	29 (28)	1.6 (0.4-5.4)	0.490
∗AB+∗BB/∗AlaAla	24 (9)	7 (7)	0.9 (0.2-3.4)	0.906
∗AB+BB/∗AlaVal+∗ValVal	121 (47)	60 (57)	2.1 (1.0-4.5)	0.056

CI: confidence interval; OR: odds ratio. ^a^Logistic regression to compute odds ratios adjusted for gender, age, smoking, hypertension, and diabetes. ^b^Reference group. ^∗^Statistically significant in comparison with the reference genotype (*p* < 0.05).

**Table:** (b) Distribution of *GSTA1*, *GSTP1*, *GSTM1*, and *GSTT1* genotypes in IDC patients and controls

Genotype	Controls (*n* (%))	Patients (*n* (%))	OR (95% CI)^a^	*p*
*GSTA1*				
∗A/A	112 (41)	30 (35)	1.0^b^	
∗A/B+B/B	162 (59)	55 (65)	1.2 (0.6-2.2)	0.660
*GSTP1*∗Ile105Val (rs1695)				
∗Ile/Ile	115 (42)	29 (34)	1.0^b^	
∗Ile/Val+∗Val/Val	159 (58)	56 (66)	1.4 (0.7-2.7)	0.284
*GSTM1*				
Active	137 (50)	39 (46)	1.0^b^	
Null	137 (50)	46 (54)	1.2 (0.6-2.2)	0.620
*GSTT1*				
Active	203 (74)	64 (75)	1.0^b^	
Null	71 (26)	21 (25)	0.9 (0.4-1.9)	0.854
Combined *GSTA1/GSTP1*				
∗AA/∗IleIle	54 (20)	13 (15)	1.0^b^	
∗AA/∗IleVal+∗ValVal	58 (21)	17 (20)	1.3 (0.5-3.6)	0.618
∗AB+∗BB/∗IleIle	61 (22)	16 (19)	1.0 (0.4-2.9)	0.924
∗AB+BB/∗IleVal+∗ValVal	101 (37)	39 (46)	1.5 (0.6-3.8)	0.348

CI: confidence interval; OR: odds ratio. ^a^Logistic regression to compute odds ratios adjusted for gender, age, smoking, hypertension, and diabetes. ^b^Reference group.

**Table 5:** Haplotype analysis of *GSTP1* polymorphisms in CAD-related HF patients and controls.

Haplotype	*GSTP1* rs4925	*GSTP1* rs156697	Controls (%)	CAD patients (%)	OR (95% CI)^a^	*p*
*GSTP1*∗A	∗A	∗C	58	59	1.0^b^	
*GSTP1*∗B	∗G	∗C	33	34	1.4 (0.9-2.3)	0.170
*GSTP1*∗C	∗G	∗T	4	4	0.5 (0.1-3.9)	0.490
*GSTP1*∗D	∗A	∗T	4	3	0.6 (0.1-3.0)	0.560

CI: confidence interval; OR: odds ratio. SNPStats was used for haplotype analysis; ^a^Logistic regression to compute odds ratios adjusted for gender, age, smoking, hypertension, and diabetes. ^b^Reference group.

**Table 6:** Markers of oxidative stress, inflammation, and endothelial dysfunction in CAD-related HF patients stratified according to GST genotypes.

Genotype	MDA (*μ*mol/L)^a^	hs-CRP (mg/L)^b^	TNF*α* (pg/mL)^b^	ICAM-1 (ng/L)^b^	VCAM-1 (ng/L)^b^
*GSTA1*					
∗A/A	7.53 ± 0.86	2.12 (0.49-43.51)	2.25 (0.15-47.63)	375.90 (206.43-696.91)	1113.12 (563.85-3697.45)
∗A/B+B/B	7.57 ± 0.73	2.14 (0.15-45.00)	2.25 (0.22-29.76)	380.98 (264.20-691.04)	1063.23 (144.45-7429.55)
	0.802	0.600	0.792	0.526	0.505
*GSTP1* (rs1695)					
∗Ile/Ile	7.53 ± 0.87	1.91 (0.38-35.68)	2.15 (0.33-11.12)	363.38 (206.43-553.84)	1152.30 (563.85-1793.90)
∗Ile/Val+∗Val/Val	7.58 ± 0.72	2.29 (0.15-45.00)	2.34 (0.15-47.63)	390.10 (255.12-696.91)	1095.20 (144.45-7429.55)
*p*	0.745	0.382	0.557	0.041	0.882
*GSTP1* (rs1138272)					
∗Ala/Ala	7.55 ± 0.79	2.14 (0.15-45.00)	2.21 (0.15-11.12)	381.25 (206.43-696.91)	1109.25 (144.45-7429.55)
∗Ala/Val+∗Val/Val	7.72 ± 0.76	2.08 (0.93-43.51)	4.69 (0.63-47.63)	351.35 (275.60-568.10)	1040.95 (759.25-1871.55)
*p*	0.436	0.777	0.045	0.152	0.899

MDA: malondialdehyde; hs-CRP: high-sensitivity C-reactive protein; TNF*α*: tumor necrosis factor-alpha; ICAM-1: intercellular adhesion molecule-1; VCAM-1: vascular cell adhesion molecule-1. ^a^Student t-test was used. ^b^Mann-Whitney test was used. ^∗^Statistically significant at *p* < 0.05.

**Table 7:** Echocardiographic and endothelial parameters stratified according to HF risk-associated GST genotypes in CAD subgroup.

Genotype	LVEDD^a^	LVESD^a^	FMD-NO^b^	FMD-NTG^b^
*GSTP1*∗Ile105Val (rs1695)				
∗Ile/Ile	6.0 ± 0.8	4.8 ± 1.0	3.8 ± 4.1	12.4 ± 7.1
∗Ile/Val+∗Val/Val	6.0 ± 0.7	4.6 ± 0.9	4.9 ± 5.1	11.3 ± 6.4
*GSTP1*∗Ala114Val (rs1138272)				
∗Ala/Ala	6.0 ± 0.8	4.7 ± 0.9	4.4 ± 4.7	11.83 ± 7.1
∗Ala/Val+∗Val/Val	6.0 ± 0.8	4.6 ± 1.1	5.51 ± 5.48	10.71 ± 5.1
Combined *GSTA1/GSTP1*(rs1695)				
∗AA/∗IleIle	6.3 ± 0.7	5.1 ± 0.8	3.8 ± 4.0	11.3 ± 7.4
∗AA/∗IleVal+∗ValVal	6.0 ± 0.7	4.6 ± 1.0	5.6 ± 4.8	11.4 ± 8.4
∗AB+∗BB/∗IleIle	5.8 ± 0.9	4.5 ± 1.1	3.8 ± 4.2	13.1 ± 7.0
∗AB+BB/∗IleVal+∗ValVal	5.9 ± 0.7	4.5 ± 0.9^∗^	4.7 ± 5.3	11.2 ± 5.4
Combined *GSTA1/GSTP1*(rs1138272)				
∗AA/∗AlaAla	6.1 ± 0.6	4.8 ± 0.8	5.2 ± 4.9	11.6 ± 8.6
∗AA/∗AlaVal+∗ValVal	6.2 ± 0.9	4.9 ± 1.2	3.6 ± 2.5	10.7 ± 5.4
∗AB+∗BB/∗AlaAla	5.9 ± 0.8	4.5 ± 0.9	3.9 ± 4.6	11.9 ± 6.2
∗AB+BB/∗AlaVal+∗ValVal	5.9 ± 1.0	4.5 ± 1.0	7.4 ± 7.3	9.5 ± 5.4

## Data Availability

The database used to support the findings of this study is available from the corresponding author upon request.
